# Immunovirological markers in HTLV-1-associated myelopathy/tropical spastic paraparesis (HAM/TSP)

**DOI:** 10.1186/s12977-019-0499-5

**Published:** 2019-11-29

**Authors:** Yoshimi Enose-Akahata, Steven Jacobson

**Affiliations:** Viral Immunology Section, National Institute of Neurological, Disorders and Stroke, National Institutes of Health, 9000 Rockville Pike, Building 10 Room 5C-103, Bethesda, MD USA

**Keywords:** HTLV-1, Neurological disease, HAM/TSP, Inflammation, Central nervous system, Cerebrospinal fluid

## Abstract

Human T cell lymphotropic virus 1 (HTLV-1) is a human retrovirus and infects approximately 10–20 million people worldwide. While the majority of infected people are asymptomatic carriers of HTLV-1, only 4% of infected people develop HTLV-1-associated myelopathy/tropical spastic paraparesis (HAM/TSP). HAM/TSP is a chronic, progressive, neurological disease which usually progresses slowly without remission, and is characterized by perivascular inflammatory infiltrates in chronic inflammatory lesions of the central nervous system (CNS), primarily affecting the spinal cord. A high HTLV-1 proviral load, high levels of antibodies against HTLV-1 antigens, and elevated concentration of proteins are detected in cerebrospinal fluid (CSF) of HAM/TSP patients. These chronically activated immune responses against HTLV-1 and infiltration of inflammatory cells including HTLV-1 infected cells into the CNS contribute to clinical disability and underlie the pathogenesis of HAM/TSP. Since the disease development of HAM/TSP mainly occurs in adults, with a mean age at onset of 40–50 years, it is important for HTLV-1-infected carriers and HAM/TSP patients to be monitored throughout the disease process. Recent advances in technologies and findings provide new insights to virological and immunological aspects in both the CNS as well as in peripheral blood. In this review, we focus on understanding the inflammatory milieu in the CNS and discuss the immunopathogenic process in HTLV-1-associated neurologic diseases.

## Background

Human T lymphotropic virus type 1 (HTLV-1) is a human retrovirus that is associated with a persistent infection in humans [1]. HTLV-1 is thought to infect 10–20 million people worldwide. While endemic areas for HTLV-1 in the world include southern parts of Japan, the Caribbean, South America, Central and West Africa, and foci in Middle east, Australia and Melanesia [2], HTLV-1 seroprevalence is still largely unknown for a number of world populations, even in neighboring regions of endemic areas. The majority of HTLV-1 infections remain asymptomatic, but small subsets of infected individuals develop a clinical disease such as adult T-cell leukemia/lymphoma (ATLL), HTLV-1 associated myelopathy/tropical spastic paraparesis (HAM/TSP), and other inflammatory disorders [3–5]. HAM/TSP is a chronic, progressive, neurological disease clinically characterized by progressive lower extremity weakness, spasticity, and bladder/bowel sphincter dysfunction [6]. The disease development of HAM/TSP mainly occurs in adults, with a mean age at onset of 40–50 years, with a higher prevalence in women than in men at a ratio of approximately 3:1 [7]. The disease usually progresses slowly without remission, but the clinical course and rate of progression may vary greatly among patients [7]. Although clinical improvements have been reported for a number of agents such as corticosteroids, currently, no therapy has been shown to significantly modify the long-term disability associated with HAM/TSP.

The central nervous system (CNS) was thought as an immune-privileged site with no lymphatic drainage but it is now recognized to mount robust immune responses to various viral infections of the CNS that is unique from the immune response in peripheral tissues. The pathogenesis of HAM/TSP has been demonstrated to involve strong inflammatory responses in the CNS, with perivascular inflammatory infiltrates in the brain and spinal cord [6]. Unlike patients with ATLL, there are some virological and immunological similarities in peripheral blood between HAM/TSP patients and HTLV-1-infected asymptomatic carriers. Therefore, findings associated with the local inflammatory milieu in the CNS may reflect immune pathology in HAM/TSP and can lead to a better understanding of disease pathogenesis, progression and clinical treatment. In this review, we summarize the immunopathogenic features of HAM/TSP, focusing on the local virological and immunological responses in the CNS, and discuss future clinical and basic research in HTLV-1-associated neurologic diseases.

## Epidemiology and lifetime risk of HAM/TSP

### Epidemiology

Before the discovery of HTLV-1, clinical observations with unusual prevalence of spastic paraplegia have been reported mainly from the Caribbean islands. In the mid 1980s, seroprevalence studies in the Caribbean islands and Japan demonstrated that HTLV-1 specific antibodies existed in high proportion of patients with the disorder, subsequently designated as HAM/TSP [3, 4]. Currently, clinical observations with HAM/TSP have been reported worldwide. The lifetime risk of HAM/TSP development has been reported as 0.25% in HTLV-1-infected individuals in a southern Japanese population whereas the risk in population of Afro-Caribbean descent has been reported as 1.9 to 2.4% and increasing to 3.7% after 10 years of follow up study [8–10]. In Central Africa, high frequency of HAM/TSP cases have been reported in northern Zaire concomitant with a high HTLV prevalence in the population [11]. A recent study of Martinique reported that the temporal trends in HAM/TSP incidence over 25 years was a significant decrease of more than 70% in the incidence of HAM/TSP in early 2000 compared to the 1986–2000 period [12]. However, in Brazil, the study based on HTLV-1-seropositive cases over 15 years reported HAM/TSP incidence rate of 5.3 cases per 1000 cases infected with HTLV-1 per year [13]. Central Australia also has a high adult prevalence of HTLV-1 infection, exceeding 40% in remote indigenous communities, with a few reported cases of patients diagnosed with HAM/TSP [14, 15].

Although Europe and North America are often considered as nonendemic areas for HTLV-1 infection, high rates of HTLV-1 infection have been reported in some regions of Europe and North America where most HAM/TSP patients originated from HTLV-1 endemic areas, such as the West Indies, Africa, Caribbean and South America [2]. Recently, a patient who diagnosed with typical HAM/TSP and was immigrated from West Africa to North America at a young age has been reported to carry a primate T lymphotropic virus-1 (PTLV-1), closely related to strains of simian T lymphotropic virus-1 (STLV-1) which is simian counterpart of HTLV-1 [16]. Since the increased global travel and immigration have contributed to the increased risk of virus transmission in human populations, a potential risk of HTLV-1-associated diseases is not only limited to populations in endemic areas. In addition, a concern in nonendemic areas is that a number of cases with chronic progressive myelopathy have often been falsely diagnosed as multiple sclerosis in which the primary progressive form is clinically similar to HAM/TSP. The reevaluation of the global burden of infection and the expansion of HTLV-1 screening policies are clearly needed both in endemic and in nonendemic areas.

### Host genetic factors

Unlike human immunodeficiency virus-1 (HIV-1), the genetic variations of HTLV-1 are minimal both within and between hosts and there is no HTLV-1 strain or sequence variants directly associated with any disease outcome [17, 18]. In addition, association of HTLV-1 infection with clinical parameters for HAM/TSP, such as the rate of disease progression, the age of onset, the gender and the history of HTLV-1 transmission, slightly vary in different geographic regions [19, 20]. Therefore, the different outcomes of an HTLV-1 infection are thought to be associated with differences in the host response to the virus rather than the virus itself. Several lifetime risks of developing HAM/TSP have been reported including human leukocyte antigen (HLA) and non-HLA gene polymorphisms. The HLA class I genotype of HTLV-1-infected individuals determines the specificity and the efficacy of CD8^+^ T cell responses to the virus, which control HTLV-1 provirus load (PVL) in the host and influence the susceptibility to HTLV-1-associated diseases. The HLA class I genes, *HLA*-A*02 and *HLA*-Cw*08, were associated with significant reduction in PVL and protective effect from HAM/TSP in Southern parts of Japan [21, 22]. A protective effect of *HLA*-A*02 was also observed in Brazil [23]. In addition, the HLA class I genes, *HLA*-A*02 and *HLA*-Cw*08, showed stronger binding of an HTLV-1 basic leucine zipper factor (HBZ) peptide, which was associated with lower HTLV-1 PVL and risk of HAM/TSP [24]. In contrast, the class I alleles, *HLA*-B*07 and *HLA*-B*5401, and the class II allele, *HLA*-DRB1*0101, were associated with a higher susceptibility to HAM/TSP [21, 22, 25].

Analysis of single nucleotide polymorphisms (SNPs) demonstrated the associations of some host genes with the outcome of an HTLV-1 infection. A polymorphism in the promoter of the immunosuppressive cytokine Interleukin-10 (IL-10: *IL10*-*592A*) was associated with a two-fold reduction in the odds of developing HAM/TSP in Japan [26]. In a study from Brazil, a polymorphism in the promoter of the inflammatory cytokine IL-6 (*IL6*-*634C*) was detected at a higher frequency in HAM/TSP patients than in asymptomatic carriers while the association of *IL10*-*592A* polymorphism was not observed [27]. In another study, the promotor polymorphism (*TNF*-*863A*) of the inflammatory cytokine tumor necrosis factor (TNF), has been reported to be also associated with the risk of HAM/TSP [28]. In contrast, polymorphism in the 3UTR of the chemokine stromal cell-derived factor 1 (SDF-1: *SDF1 *+ *801A*) and IL-15 *(IL*-*15 *+ *191C)* were associated with reduction in the risk of developing HAM/TSP [28]. Analysis of genetic variants of host restriction factors in HAM/TSP patients demonstrated that TRIM5α polymorphisms might also be associated with HTLV-1 PVL, but no specific mutation of host restriction factors was observed in HAM/TSP patients [29]. These observations again support the hypothesis that host genetic factors play an important role in control of HTLV-1 infection or immune regulation of HTLV-1-infected individuals and may be influenced by ethnicity and environmental factors within geographic regions.

## Mechanism of HAM/TSP development

### Neuropathology

Early in the course of the disease, the inflammatory infiltrate contains equal numbers of CD4^+^ T cells, CD8^+^ T cells, and foamy macrophages in the spinal cords of HAM/TSP patients. Over time, CD8^+^ T cells are predominantly detected in the chronic inflammatory lesions of patients with longer duration of disease [6, 30]. In HAM/TSP patients with active-chronic inflammation, perivascular inflammatory infiltration were seen in the brain as well as in the spinal cord [30]. Intrathecal HTLV-1-specific antibody production provides additional data to support the diagnosis of HAM/TSP [31]. In HAM/TSP patients, mild lymphocyte pleocytosis in cerebrospinal fluid (CSF) was detected in approximately one-third of cases as well as mildly elevated concentration of protein in the CSF [32, 33]. The inflammatory process has been visualized and quantified by magnetic resonance imaging (MRI) to be shown as the loss of spinal cord volume, suggesting the destructive pathological processes in HAM/TSP, such as irreversible demyelination and loss of astroglia, neuronal cell bodies, and axons [34–36]. A recent longitudinal study of spinal cord cross-sectional area measurements showed that spinal cord atrophy began in the thoracic cord and progressed to the cervical cord in HAM/TSP patients with rapid progression [34]. Interestingly, a more atrophic spinal cord in HAM/TSP was associated with higher percentage of inflammatory CD8^+^ T cells and HTLV-1 PVL in CSF of HAM/TSP patients [34]. Thus, chronically activated immune responses against HTLV-1 and infiltration of inflammatory cells including HTLV-1 infected cells into the CNS contribute to clinical disability and underlie the pathogenesis of HAM/TSP.

### HTLV-1 infection and expressions

HTLV-1 PVL in PBMCs varies widely between individuals and remains relatively stable within individuals over time. As a group, HAM/TSP patients have typically higher HTLV-1 PVL than asymptomatic carriers [37], although longitudinal follow-up studies showed that a significant number of asymptomatic carriers can have high HTLV-1 PVL in the PBMCs for long periods of time without developing clinical symptoms associated with HTLV-1 infection [38, 39]. Importantly, HTLV-1 PVL has been reported to be higher in the cells of CSF, than in the matched PBMCs of HAM/TSP patients by approximately threefold [38, 40–43]. In addition, HAM/TSP patients had significantly higher HTLV-1 PVL in the CSF, compared to asymptomatic carriers and HTLV-1-infected individuals with other neurologic diseases [41, 44]. Higher ratio of HTLV-1 PVL in the CSF to that in the PBMCs was significantly associated with clinically progressive disease and with recent onset of HAM/TSP [43]. These findings suggested that it is important to monitor the HTLV-1 PVL as a biomarker associated with the inflammatory milieu in the CNS that may serve to predict disease progression in HTLV-1 infected individuals.

The HTLV-1 proviral genome has structural genes, *gag*, *pol*, and *env* flanked by long terminal repeat at both ends. HTLV-1 genome also contains a *pX* region between *env* and 3′ LTR encoded several accessory genes including *tax* and *HBZ* [45]. The viral genes are transcribed from the 5′ LTR, but only *HBZ* encoded on the minus strand of the provirus is transcribed from the 3′ LTR. Two HTLV-1 genes, *tax* and *HBZ*, have been shown to play important roles in the pathogenesis of HAM/TSP. Tax is a transforming and transactivating protein of HTLV-1 and induces the expression of a variety of cellular genes by activation of the NF-kB and CREB/ATF pathways [45]. Although HTLV-1 *tax* mRNA and Tax protein are rarely or undetectable directly in fresh PBMCs of HTLV-1-infected individuals, HAM/TSP patients showed a spontaneous increase of *tax* mRNA and Tax protein expression in PBMCs after ex vivo culture without any exogenous stimulators. This observation peaks at 12–24 h and is significantly higher in HAM/TSP patients than in asymptomatic carriers [46, 47]. In addition, HTLV-1 *tax* mRNA and Tax proteins have been reported to be detected in CSF cells and within spinal cord and cerebellar sections of HAM/TSP patients [48–50]. The increased expression of HTLV-1 Tax protein in the CSF cells was more frequent in HAM/TSP patients with shorter duration of illness [48]. These finding suggested that the presence of Tax protein in the CNS might cause direct cell damage in the nervous system and may serve to activate and generate Tax-specific immune responses in HAM/TSP patients. Unlike *tax* gene products, *HBZ* mRNA is ubiquitously expressed in HTLV-1-infected cells and promotes the growth and survival of the leukemic cells [45]. The expression of *HBZ* mRNA was detected in PBMCs of HAM/TSP patients, which was significantly lower than in ATL patients but higher than in asymptomatic carriers [51]. In contrast to *HBZ* mRNA, HBZ protein has been reported to be rarely detected in HAM/TSP patients, but recent reports demonstrated that HBZ protein was localized in the cytoplasm of CD4^+^ T cells, irrespective of co-expression of CD25 [52]. Since *HBZ* mRNA in PBMCs was correlated with disease severity in HAM/TSP patients [51], it is of interest how *HBZ* gene products could be associated with CNS inflammation and damage in HAM/TSP patients.

HTLV-1 infection is considered to be latent in the infected individuals. However, the presence of chronically activated HTLV-1-specific immune responses suggested that HTLV-1 antigens might be continuously synthesized. Comparisons of HTLV-1 integration sites between infected individuals revealed that HTLV-1 integration might be more frequent in transcriptionally active areas of the genome in HAM/TSP patients than in asymptomatic carriers, which was associated with an increased rate of Tax expression [53]. Moreover, a larger number of unique insertion sites was detected in HAM/TSP patients than in asymptomatic carriers whereas there was no significant differences in oligoclonality of HTLV-1 integration between HAM/TSP patients and asymptomatic carriers [54]. The targets of integration were strongly associated with the presence of a binding site for specific host transcription factors, such as p53, HDAC6 and STAT1. The presence of the chromatin remodeling factors BRG1 and INI1 and certain host transcription factors either upstream or downstream of the provirus was associated with silencing or spontaneous expression of the provirus, respectively [55]. A recent report revealed that CTCF, a zinc-finger protein and a key regulator of chromatin structure and function, bound to HTLV-1 and formed loops between HTLV-1 proviral and host genes to regulate HTLV-1 transcription and RNA splicing [56]. Interestingly in HAM/TSP, the majority of spontaneous Tax expressing cells corresponded to a large number of low abundance clones, rather than a small number of high abundance clones [55]. These findings suggested that interference of host gene transcription and chromatin remodeling may be critical determinants of proviral latency in natural HTLV-1 infection whereas clonal expansion of infected cells might be controlled by host immune responses to Tax or by other viral factors such as HBZ in HAM/TSP patients. Future research will address how and where expression of HTLV-1 genes are regulated in HTLV-1-infected individuals.

As described above, regardless of the absence of viral RNA and proteins in fresh PBMCs, spontaneous increase in plus-strand HTLV-1 transcription was detectable when PBMCs of HTLV-1 infected individuals are cultured ex vivo. The question whether extracellular microenvironment can contribute to regulation of HTLV-1 expression remains unknown. Interestingly, a recent report demonstrated that physiological hypoxia significantly enhanced HTLV-1 reactivation from latency whereas inhibition of glycolysis or the mitochondrial electron transport chain suppressed HTLV-1 plus-strand transcription ex vivo [57]. This may have clinical consequences since brain, the highest consumer of oxygen, is likely to have an increased risk of hypoxia-induced neurological damage, which has been suggested to be associated with age in many CNS diseases such as stroke, Alzheimer’s disease and encephalopathy [58]. The glucose receptor GLUT-1 has been reported to be one of the cellular receptors for HTLV-1 and the expression of GLUT-1 is induced by hypoxia [57, 59]. These findings suggested that glucose metabolism and oxygen availability might play an important role in regulation of the latency, reactivation and productive infection of HTLV-1. It is also of interest that HTLV-1 *tax* gene and *tax* mRNA was detected in the bone marrow of HAM/TSP patients, which is also physiologically hypoxic [60]. Thus, the extracellular microenvironment in CNS tissues may be an important contributing factor that may initiate a series of pathophysiological events leading to clinical disease.

## CSF cellular immune responses in HAM/TSP

### CD4^+^ T cell

#### A reservoir of HTLV-1

CD4^+^ T cells are the predominant reservoir of HTLV-1. In HAM/TSP patients, CD4^+^CD25^+^ T cells contain high frequency of HTLV-1 proviral DNA, express HTLV-1 *tax* mRNA at significantly higher levels than in CD4^+^CD25^−^ T cells and produce various cytokines including IFN-γ [61]. CD4^+^CD25^+^ T cells were significantly higher in the CSF as well as in peripheral blood of HAM/TSP patients, compared to healthy controls and asymptomatic carriers, which was also significantly correlated with HTLV-1 PVL in the CSF of HAM/TSP patients [62]. Furthermore, CD25^+^CCR4^+^CD4^+^ T cells have high HTLV-1 PVL and are associated with functional changes including high production of IFN-γ in HAM/TSP patients, which was found to be correlated with disease activity and severity [61, 63, 64]. Abundant CD4^+^CCR4^+^ T cells which co-expressed the Th1 marker CXCR3 and produced T-bet and IFN-γ were also present in CSF and spinal cord lesions in HAM/TSP [63]. IFN-γ producing HTLV-1-infected CD4^+^ T cells stimulated astrocytes to secrete the chemokine CXCL10 (IP-10), a ligand of CXCR3 [65] and suggest that CXCL10 from astrocytes might recruit additional HTLV-1-infected CXCR3^+^CD4^+^ T cells into the CNS of HAM/TSP patients.

#### Dysregulation

Regulatory CD4^+^ T cells (Treg) that constitutively express CD25 (the IL-2 receptor α chain) are engaged in the maintenance of immunologic self-tolerance by suppressing the activation and expansion of self-reactive lymphocytes that may cause autoimmune diseases [66]. However, Tax protein downregulates forkhead box P3 (FoxP3) in CD4^+^CD25^+^ T cells due to decreased demethylation of the Foxp3 gene, which caused the decreased suppressive capacity of CD4^+^CD25^+^ T cells and stimulation of HTLV-1 Tax-specific CD8^+^ T cells in HAM/TSP patients [64, 67, 68]. Other immune molecules related to Treg were also dysregulated in HAM/TSP patients. Transforming growth factor-β (TGF-β), play critical roles in suppressing the immune response, such as inhibition of proinflammatory responses and promotion of Treg generation and function. In HAM/TSP, HTLV-1 Tax inhibited TGF-β RII and Smad7 expression resulting in dysregulation of TGF-β signaling [69]. Helios, a member of the Ikaros family of transcription factors, is highly expressed in human Treg, but HAM/TSP patients showed decreased Helios expression and enhanced cell adhesion molecules in CD4^+^ T cells [70]. A coinhibitory molecule, cytotoxic T-lymphocyte-associated protein 4 (CTLA-4), was also downregulated in peripheral blood CD4^+^CD25^+^ T cells of HAM/TSP patients [62].

#### Cell adhesion molecules

The CNS is protected from the entry of pathogens, circulating immune cells, and factors within the blood by physiological structure called the blood–brain barrier (BBB) which is maintained by the endothelial cells of cerebral microvessels with tight junctions. CD4^+^ T cells are routinely exposed in vivo to alterations in the microenvironment, which was associated with enhanced production of various soluble factors as well as expression of cell adhesion molecules due to activation of HTLV-1 expression. Since interaction of cell adhesion molecules induces recruitment and extravasation of lymphocytes through the BBB, increased expression of cell adhesion molecules may facilitate the migration of HTLV-1-infected lymphocytes across the BBB endothelium. Tax has been demonstrated to regulate cell adhesion molecules, such as intercellular adhesion molecule-1 (ICAM-1), vascular cell adhesion molecule 1 (VCAM-1) and cell adhesion molecule 1 (CADM1/TSLC1) in HAM/TSP patients [71–73]. CADM1^+^CD4^+^ T cells expressed higher coinhibitory molecule, T cell immunoglobulin and ITIM domain (TIGIT), in HAM/TSP patients compared to healthy controls [74]. Activated leukocyte cell adhesion molecule (ALCAM/CD166), a member of the immunoglobulin superfamily, is overexpressed on the surface of HTLV-1-infected lymphocytes, both in chronically infected cell lines and in primary CD4^+^CD25^+^ T cells from HAM/TSP patients [75].

Thus, CD4^+^ T cells are an important immune population that serve as the major reservoir of HTLV-1 due to HTLV-1 expression and can also have dynamic functional changes including cell migration, activation and dysregulation.

### CD8^+^ T cell

#### HTLV-1-specific CD8^+^ T cells

Tax is an immunodominant antigen recognized by HTLV-1-specific cytotoxic CD8^+^ T cells (CTLs) [76]. CD8^+^ T cells play a crucial role in immunity against HTLV-1 through their ability to secrete various factors that suppress viral replication and kill infected target cells in HTLV-1-infected subjects [77, 78]. However, while CD4^+^ T cells are more prevalent than CD8^+^ T cells in CSF lymphocytes of healthy individuals, in the CSF of HAM/TSP patients there was an increased predominance of CD8^+^ T cells over CD4^+^ T cells [79]. Importantly, the frequency of HTLV-1 Tax-specific CD8^+^ T cells was higher in CSF than in peripheral blood, and was correlated with HTLV-1 PVL [42, 80, 81]. It has been demonstrated that HTLV-1 Tax-specific CD8^+^ T cells as well as CD4^+^ T cells expressing HTLV-1 Tax proteins were detected in the parenchyma of HAM/TSP spinal cords, suggesting that the interaction between HTLV-1-specific CTLs and HTLV-1-infected CD4^+^ T cells may cause bystander damage to resident cells in the CNS [82]. In contrast to HTLV-1 Tax specific CD8^+^ T cells, a low frequency of HTLV-1 HBZ-specific CD8^+^ T cells are detected in peripheral blood of asymptomatic carriers and HAM/TSP patients, and HBZ-specific CTL clones were able to lyse naturally infected cells [24]. However, the binding affinity of HBZ peptides to HLA class I molecules was found to be significantly weaker than that of peptides from Tax [24, 83]. It remains to be determined as to the significance and role that HTLV-1 HBZ-specific CD8^+^ T cells play in the pathogenesis of HAM/TSP.

#### Expansion of CD8^+^ T cells

One of the most striking features of the cellular immune response in HAM/TSP patients is the increased numbers of memory and/or effector CD8^+^ T cells and HTLV-1 Tax-specific cytotoxic CD8^+^ T cells. The common γ chain family of cytokines including IL-2, IL-7, IL-9, IL-15, and IL-21 play an important role in lymphocyte proliferation, survival and function during immune responses and homeostasis. Tax has been shown to transactivate a number of the common γ chain family of cytokines and the receptors, such as IL-2/IL-2R, and IL-15/IL-15R [84]. Since both IL-2 and IL-15 induce the proliferation and increase the cytolytic activity of CD8^+^ T cells, it has been suggested that IL-2/IL-2R and IL-15/IL-15R autocrine loop may contribute to the pathogenesis of HAM/TSP [85]. A subset of memory CD8^+^ T cells, stem cell-like memory T cells (Tscm), has been reported to be identified as a naïve phenotype but express increased level of CD95, IL-2Rβ, CXCR3 and LFA-1 and have similar functions to memory T cells including the ability to proliferate rapidly and release inflammatory cytokines in response to antigen re-exposure [86]. Recently, it has been demonstrated that the frequency of Tscm was significantly increased in HAM/TSP patients compared to healthy controls, suggesting that an adequate number of functionally competent memory CD8^+^ T cells might be sustained through cytokine-driven homeostatic proliferation to achieve long-lived protection against chronic HTLV-1 infection [87]. Using a new high throughput sequencing technology, analysis of T cell receptor (TCR) repertoire recently revealed that HAM/TSP patients showed a higher clonal T cell expansion in peripheral blood compared to patients with multiple sclerosis and healthy controls [88]. Since the TCR clonal repertoire in the peripheral blood was different from that in the CSF, further studies are required to determine T cell profiles including clonality, diversity and commonality in CSF of HAM/TSP patients.

#### Effector function of CD8^+^ T cells

During chronic viral infection, antigen-specific CD8^+^ T cells initially acquire effector functions but gradually become less functional as the infection progresses. In HTLV-1 infection, although HTLV-1-specific CTL responses were detected in both asymptomatic carriers and HAM/TSP patients, high expression of IFN-γ in CD8^+^ T cells specifically in HAM/TSP patients compared to asymptomatic carriers have been reported to be induced by interaction with virus-infected CD4^+^ T cells and CD8^+^ T cells [89–91]. Interestingly, CD8^+^ T cells in patients with HAM/TSP, but not in asymptomatic carriers, were demonstrated to spontaneously degranulate and produce IFN-γ [92]. In HAM/TSP patients, expression of *IL*-*15* mRNA and IL-15 protein is up-regulated in non-T cells and CD14^+^ monocytes more than those in healthy controls or asymptomatic carriers [85, 92]. In addition, HTLV-1 Tax-specific CD8^+^ T cells showed higher expression of IL-15Rα, compared with CMV pp65-specific CD8^+^ T cells [93]. It was later supported by a report that IL-15 stimulated HTLV-1 Tax-specific CD8^+^ T cells, but not CMV pp65-specific CD8^+^ T cells, to induce degranulation and IFN-γ expression [92]. Thus, the increase of the common γ chain family of cytokines and receptors in HAM/TSP patients may be involved in increased proliferation and enhanced cytolytic activity and inflammatory cytokine production of HTLV-1-specific CD8^+^ T cells.

#### Costimulatory and coinhibitory molecules

CD8^+^ T cell responses during chronic viral infection are regulated by complex patterns of co-expressed stimulatory and inhibitory receptors. A number of costimulatory and coinhibitory receptors has been reported to be involved with HAM/TSP. The expression of CD244 (2B4), a signaling lymphocyte activation molecule (SLAM) family receptor, was significantly higher on CD8^+^ T cells in both asymptomatic carriers and HAM/TSP patients, than those on healthy controls [94]. High CD244 expression was demonstrated on HTLV-1-specific CD8^+^ T cells as well as CMV-specific CD8^+^ T cells in a patient with HAM/TSP. Importantly, SLAM-associated protein (SAP) which play a role in controlling the active transduction was overexpressed in HAM/TSP patients compared to asymptomatic carriers and healthy controls whereas there was no significant differences of expression of EAT-2, a SLAM-related inhibitory protein. Blockade of CD244 and SAP inhibited degranulation and IFN-γ production in CD8^+^ T cells of HAM/TSP patients, suggesting that CD244/SAP pathway might be involved in the active regulation of HTLV-1-specific CD8^+^ T cells of HAM/TSP patients [94]. Furthermore, T cell immunoglobulin and mucin domain-containing protein 3 (Tim-3) was reduced on CD8^+^ T cells and HTLV-1-specific CD8^+^ T cells of HAM/TSP patients [95, 96]. Although expression of programmed death receptor-1 (PD-1) has been reported in peripheral blood T cells in HAM/TSP compared to asymptomatic carriers and/or healthy controls, studies have varied [95–98]. A recent report demonstrated that, when the frequency of PD-1^+^ T cells were compared among healthy controls, patients with HAM/TSP and Progressive Multifocal Leukoencephalopathy (PML) which is a rare, often fatal, demyelinating disease caused by reactivation of the ubiquitous JC virus, HAM/TSP patients as well as PML patients showed a significant higher percentage of PD-1 expression on CD4^+^ and CD8^+^ T cells in CSF than healthy controls [97]. Although the relative contribution of the above mentioned costimulatory and coinhibitory factors to the observed dysregulation of chronically activated HTLV-1-specific CD8^+^ T cells in HAM/TSP patients remains to be determined, restoration or modulation of virus-specific CD8^+^ T cells in HAM/TSP patients may be important to prevent further tissue damage in the CNS and progression of HAM/TSP.

### B cell

#### Humoral response

Intrathecal antibody synthesis is a well-documented phenomenon in infectious and demyelinating neurological diseases. As definitive laboratory diagnosis of HAM/TSP is based on the presence of anti-HTLV-1 antibodies in the blood and CSF. Robust humoral immune responses against HTLV-1 antigens have been reported in peripheral blood and CSF of HAM/TSP patients. Intrathecal antibody synthesis against HTLV-1 has been demonstrated, as evidenced by the presence of HTLV-1-specific antibodies and oligoclonal IgG bands in CSF of patients [31]. Intrathecal antibody response to HTLV-1 inversely correlates with higher HTLV-1 PVL and a worse prognostic outcome [99]. In addition, antibodies against two HTLV-1 viral products, Tax and Gag p24, have been reported to cross-react with host antigens, heterogeneous ribonucleoprotein A1 and peroxiredoxin-1, respectively, suggesting that molecular mimicry may also play a role in the pathogenesis of HAM/TSP [100, 101].

#### Antibody secreting B cell

Regardless of the presence of HTLV-1-specific antibodies and oligoclonal IgG bands in CSF of HAM/TSP patients, little is known about the CNS microenvironment related to this increased humoral immune response in HTLV-1-infected individuals. In vivo, T cells including both CD4^+^ and CD8^+^ T cells were detected in spinal cords of HAM/TSP patients and the frequency of these T cell populations varied depending on the duration of illness. B cells were only rarely observed in HAM/TSP spinal cords by in situ histopathological studies [6]. A recent report demonstrated that the B cell/monocyte ratio and antibody secreting B cells were elevated in CSF of HAM/TSP patients as well as patients with relapse-remitting multiple sclerosis compared to healthy volunteers [62]. Antibody secreting B cells including plasmablast and plasma cells are differentiated from memory B cells and maintain immunoglobulin production. Increased antibody secreting B cells in CSF of HAM/TSP patients significantly correlated with intrathecal anti-Gag antibody synthesis [62]. By comparison, in the CSF of asymptomatic carriers, B cell frequencies and the B cell/monocyte ratio was low. Moreover, antibody secreting B cells were undetectable in the CSF of asymptomatic carriers, suggesting that B cell recruitment and/or differentiation may be present in CSF of patients with neuroinflammatory diseases, but not in asymptomatic carriers. In addition, antibody secreting B cells may not be directly involved in the disease progression of HAM/TSP patients even though they may be present for long periods in the CSF. Interestingly, elevated CD4^+^CD25^+^ T cells significantly correlated with antibody secreting B cells and HTLV-1 PVL in the CSF of HAM/TSP patients [62]. These findings suggested that increased expressions of cytokines, such as IL-2, IL-15 and IL-21, from activated T cells might accelerate B cell function in HAM/TSP patients. In addition, memory follicular helper CD4^+^ T cells (Tfh), which promote B cell growth, differentiation and class switching, were decreased in the CSF of HAM/TSP patients [62]. B cell function induced independent with Tfh cells may also be associated with impaired B cell responses and generation of antigen-specific antibodies with low specificity and function.

## CSF biomarkers in HAM/TSP

### HTLV-1-specific antibodies

As HTLV-1-specific antibody responses have been associated with the pathogenesis of HTLV-1-related diseases, strong antibody responses against HTLV-1 antigens have been reported in both serum and CSF of HAM/TSP patients. Analysis of HTLV-1 specific antibodies in CSF of HAM/TSP against synthetic peptides of HTLV-1 Gag and Env proteins demonstrated that a diverse intrathecal immune response to several HTLV-1 synthetic peptides, most frequently against Gag p19 (100–130), Env gp21 (458–488), and Env gp46 (175–199 and 288–317) [102, 103]. Moreover, common peptide motifs highly homologous to HTLV-1 Env gp46 peptides (192–199 and 237–243) were frequently detected in the CSF of HAM/TSP patients [104]. HTLV-1 Tax specific antibody was also elevated in HAM/TSP CSF. When compared to antibody responses for HTLV-1 Gag, Env and Tax in CSF, intrathecal anti-Gag and anti-Tax antibody synthesis are significantly elevated in HAM/TSP patients compared to those in asymptomatic carriers [62]. Antibody response against HBZ was detected in peripheral blood of HTLV-1-infected individuals, but only in a subset of HTLV-1-infected individuals including asymptomatic carriers, patients with HAM/TSP and ATL contained anti-HBZ antibody and the antibody response against HBZ did not discriminate between clinical status [105]. In addition, antibody responses against HBZ was detectable in the CSF of HAM/TSP patients, but was not dramatically elevated, suggesting that anti-HBZ antibody is not intrathecally synthesized [105].

### Soluble proteins

Other than HTLV-1-specific antibodies, several useful or prognostic biomarkers have been reported in HAM/TSP patients. Neopterin, a derivative of pyrimidine metabolism and a useful marker of activated monocytes and macrophages, has been reported to be elevated in CSF of HAM/TSP patients [106]. The concentration of neopterin in CSF has been shown to be associated with HTLV-1 PVL in PBMCs, anti-HTLV-1 antibody and the severity of the clinical symptoms [33, 37]. Previous studies have shown increased levels of soluble Fas in CSF and serum of HAM/TSP patients as well as of multiple sclerosis [107]. OX40 is a member of the TNF receptor family that is expressed primarily on activated CD4^+^ T cells and promotes the development of effector and memory T cells. Higher levels of soluble OX40 was detected in the CSF of HAM/TSP patients with rapid progression, and OX40 was overexpressed in spinal cord infiltrating mononuclear cells in a clinically progressive HAM/TSP patient with a short duration of illness [108].

### Cytokines and chemokines

HTLV-1 Tax protein directly induces upregulation of various cytokines/chemokines. Increased concentrations of IL-1β, IL-6, GM-CSF and IFN-γ has been reported in HAM/TSP CSF [109–111]. In addition, TNF-α^+^ cells have been also detected in the CSF of HAM/TSP patients [112]. In the spinal cords, IL-1β, TNF-α, and IFN-γ were expressed on perivascular infiltrating macrophages, astrocytes and microglia in active, chronic inflammatory lesions in HAM/TSP patients with a shorter duration of illness [113].

Some chemokines, such as CXCL9, CXCL10, CCL3, CCL5 and CCL11, have been reported to be elevated in the CSF of HAM/TSP compared to those of HTLV-1 infected individuals or other noninflammatory neurologic diseases [65, 114–116]. Immunohistochemical study revealed that a larger number of CXCL10^+^ astrocytes were detected in the spinal cord lesions of patients with HAM/TSP than in control patients, suggesting that in the HAM/TSP spinal cords, astrocytes are the main producers of CXCL10 [65]. In addition, analysis of a total of 26 biomarkers candidates in blood and CSF of HTLV-1 infected individuals and HAM/TSP patients demonstrated that CXCL10, CXCL9 and neopterin in CSF were the most strongly correlated with rate of disease progression of HAM/TSP [115]. Following the study, a new proposal of classification criteria for disease activity of HAM/TSP has been reported based on clinical score and the level of neopterin and CXCL10 in the CSF [117]. Changes in CSF composition due to local activation, and drainage of meningeal immune cells could potentially provide signals to the periphery to induce T cell recruitment. Reliable biomarkers may contribute to predicting the development of HAM/TSP and improving treatment algorithms for HAM/TSP.

### Exosomes

Recent evidences demonstrate that extracellular vesicles, including exosomes, play critical roles in viral pathogenesis and control of host immune responses to viral infection. These microvesicles contain host and viral components, including proteins, mRNA, and microRNA [118]. HTLV-1 has been shown to incorporate Tax protein, viral mRNA transcripts, proinflammatory mediators into shed exosomes [119]. In HAM/TSP patients, the exosomes containing HTLV-1 Tax proteins can be excreted from CD4^+^CD25^+^ T cells ex vivo and sensitize target cells for lysis of HTLV-1 specific cytotoxic CD8^+^ T cells [120]. Importantly, exosomes containing Tax protein were detected in the CSF of HAM/TSP patient, despite the absence of viral detection in the CSF supernatant [120]. These findings suggest that incorporation of viral proteins and mRNAs into exosomes or alteration of host contents of immune cell derived exosomes may represent a mechanism by which viral antigens could be transported to the CNS and be associated with axonal degeneration and virus-specific immune responses in HAM/TSP.

## Future challenges

### Importance of screening and prevention of HAM/TSP

Recently, new evidences are accumulating that a proportion of HTLV-1-infected individuals also have neurological symptoms without fulfilling criteria for HAM/TSP. It has been reported that early neurological disorders were present in 24% of HTLV-1-infected individuals who were initially considered asymptomatic. These patients had sufficient signs and symptoms to classify them in a novel category of disease, called intermediate syndrome [121]. In addition, some clinical conditions, neurological findings and HTLV-1 PVL may be associated with further development of full-blown HAM/TSP, in individuals considered free of the disease according to currently used criteria for its diagnosis [121]. Moreover, several neurological manifestations that are not explained by HAM/TSP have been also described in HTLV-1-infected individuals, such as peripheral polyneuropathy, myositis, dysautonomia and cognitive alterations, as well as neuropathies, movement disorders and an amyotrophic lateral sclerosis (ALS)-like syndrome [122]. In rare cases, an ALS-like syndrome can occasionally be caused by retroviruses such as HIV, and has been also reported in HTLV-1-infected individuals and HAM/TSP patients [123]. Although case of HAM/TSP patients with ALS-like syndrome differed from idiopathic ALS by the presence of bladder dysfunction, sensory and autonomic symptom, and the extremely slow progression [124, 125], it is important to further understand these neurological manifestations as part of the HTLV-1-associated neurologic complex. In Central Australia, it has been recently reported a case of isolated neurogenic bladder without features of HAM/TSP, caused by HTLV-1 infection in an Aborigine renal allograft recipient [126]. Since HTLV-1 infection is associated with a variety of clinical manifestations in patients who either do not have or who did not have fully developed HAM/TSP yet, it is important for HTLV-1-infected carriers and HAM/TSP patients to be monitored for risk markers particularly at the early stages of disease.

### Animal models for HAM/TSP

Animal models provide a useful tool for the studies of infection, pathogenesis, treatment and prevention. Various stages of HTLV-1 infection and disease development have been studied using several animal models including naturally infected nonhuman primates and experimental animals such as rabbits, rats and mice [127]. Mouse models in HTLV-1 research including immune competent, immune deficient, transgenic and humanized mouse have been successfully used to study persistent HTLV-1 infection, the role of HTLV-1 accessory genes and the development of ATL. While there is no suitable small animal model to explore the pathogenesis of HTLV-1 leading to the development of HAM/TSP, certain strains of rats (HTLV-1-infected Wistar-King-Aptekman strain of rats) have been reported to produce HTLV-1-specific antibody responses and developed spastic paraparesis of the hind legs with degenerative thoracic spinal cord and peripheral nerve lesions [128–130]. HTLV-1 infected rats also demonstrated detection of HTLV-1 DNA in lesion-associated microglia and macrophage, activation of HTLV-1 pX and TNF-α mRNA and expression of IFN-γ, altered expression of apoptosis regulating genes in spinal cord lesions [131–135]. A recent paper reported that Balb/c-Rag1-hu^−/−^γc^−/−^ (Rag1) and Bone marrow Liver Thymic (BLT) mouse models for engraftment of human CD34^+^ hematopoietic stem cells demonstrated susceptibility to HTLV-1 infection with the presence of Tax in spleen and the CNS [136]. However, to date, animal models for human neurologic diseases associated with HTLV-1 infection is still limited. The continued development of small animal models would greatly facilitate studies of HTLV-1 chronic infection and pathogenesis of HTLV-1 associated neurologic diseases.

### Therapeutic target for HAM/TSP

To date, several trials of antiretroviral drugs and immunomodulatory therapies have been reported in HAM/TSP. Corticosteroids are most commonly used for therapy of HAM/TSP patients, which showed some beneficial effects, such as reduction of inflammation at early stage and improvement of motor disability [137]. The two type 1 interferons, IFN-α and IFN-β1a, were previously used in trials for HAM/TSP [138, 139]. IFN-β1a therapy reduced the expression of HTLV-1 *tax* mRNA, the frequency of HTLV-1-specific CD8^+^ T cells and spontaneous lymphoproliferation. Although HTLV-1 PVL remained stable, some measures of motor function were improved [139]. A number of studies has been reported about the effects of IFN-α, the role of IFN-α and its long-term benefit in HAM/TSP has not been conclusively shown. Unfortunately, antiretroviral drugs such as reverse transcriptase inhibitor did not show any significant effects against HTLV-1. In contrast to antiretroviral drugs, humanized monoclonal antibodies mainly targeted for the selective removal of HTLV-1-infected and activated CD4^+^ T cells have been used and demonstrated some improvements in HAM/TSP patients. A humanized monoclonal antibody against IL-2 receptor α chain (anti-Tac) demonstrated reductions of HTLV-1 PVL in peripheral blood and spontaneous lymphoproliferation in HAM/TSP patients [140]. A recent report demonstrated that a humanized anti-CCR4 monoclonal antibody (mogamulizumab) decreased the number of HTLV-1-infected cells in peripheral blood and the level of inflammatory markers, such as CXCL10 and neopterin, in CSF. Importantly, reduction in spasticity and motor disability was observed in 79% and 32% of HAM/TSP patients, respectively [141]. A humanized anti-IL-2/IL-15 receptor β chain (Hu-Mikβ1), mainly targeted to inflammatory CD8^+^ T cells, demonstrated inhibition of aberrant CD8^+^ T cell functions including spontaneous lymphoproliferation and degranulation and IFN-γ expression [87].

While not an exhaustive review of all trials in HAM/TSP, no therapy has been shown to dramatically clear HTLV-1 infection and significantly modify the long-term disability associated with HAM/TSP. The failure to detect any clinical improvement after therapy may be due to the long disease duration in HAM/TSP patients. After such prolonged periods of time, the neurological damages may not be reversible. In addition, the progression rate of HAM/TSP varies widely among patients. Therefore, early diagnosis and prompt treatment are necessary for successful disease prevention and long-term improvement of motor disability and quality of life for HAM/TSP patients.

## Conclusion

Regulation of the local immune response is crucial in protecting the CNS from viral infection and immunopathologically mediated tissue damage. Characterization of HTLV-1 infection and CSF immune responses that are associated with a neuroinflammatory milieu may provide evidence for a pathogenic signature of an immunopathogenic process in HAM/TSP. These findings may contribute to identifying biomarkers that could detect disease progression in the early stages in HTLV-1-infected individuals. Elimination of HTLV-1 infection and control of HTLV-1 reactivation from latency remain a goal for HAM/TSP. Symptomatic management directed at the immune response to HTLV-1 is also important for patients with HAM/TSP.

## Data Availability

Not applicable.

## Abbreviations

HTLV-1: human T cell lymphotropic virus 1
HAM/TSP: HTLV-1-associated myelopathy/tropical spastic paraparesis
CNS: central nervous system
CSF: cerebrospinal fluid
ATLL: adult T cell leukemia/lymphoma
HLA: human leukocyte antigen
PVL: proviral load
HBZ: HTLV-1 basic leucine zipper factor
IL: interleukin
TNF: tumor necrosis factor
Treg: regulatory CD4^+^ T cells
FoxP3: forkhead box P3
TGF: transforming growth factor
BBB: blood–brain barrier
CTL: cytotoxic CD8^+^ T cells
Tscm: stem cell-like memory T cells
TCR: T cell receptor
SLAM: signaling lymphocyte activation molecule
SAP: SLAM-associated protein
PD-1: programmed death receptor-1
PML: progressive multifocal leukoencephalopathy
Tfh: follicular helper CD4^+^ T cells
